# Sulfated-Polysaccharide Fraction from Red Algae G*racilaria caudata* Protects Mice Gut Against Ethanol-Induced Damage

**DOI:** 10.3390/md9112188

**Published:** 2011-11-02

**Authors:** Renan Oliveira Silva, Geice Maria Pereira dos Santos, Lucas Antonio Duarte Nicolau, Larisse Tavares Lucetti, Ana Paula Macedo Santana, Luciano de Souza Chaves, Francisco Clark Nogueira Barros, Ana Lúcia Ponte Freitas, Marcellus Henrique Loiola Ponte Souza, Jand-Venes Rolim Medeiros

**Affiliations:** 1 LAFFEX—Laboratory of Experimental Physiopharmacology, Biotechnology and Biodiversity Center Research (BIOTEC), Federal University of Piauí-CMRV, 64202-020, Parnaíba, PI, Brazil; E-Mails: renan.oliveira25@yahoo.com.br (R.O.S.); geicysantos2009@hotmail.com (G.M.P.d.S.); lucasnicolau5@hotmail.com (L.A.D.N.); 2 LAFICA—Laboratory of Pharmacology of Inflammation and Cancer, Department of Physiology and Pharmacology, Federal University of Ceará, 60430-270, Fortaleza, CE, Brazil; E-Mails: larisselucetti@hotmail.com (L.T.L.); apmacedo1@hotmail.com (A.P.M.S.); souzamar@ufc.br (M.H.L.P.S.); 3 Laboratory of Proteins and Carbohydrates of Marine Algae, Department of Biochemistry and Molecular Biology, Federal University of Ceará, 60455-760, Fortaleza, CE, Brazil; E-Mails: lucianoscsep@hotmail.com (L.d.S.C.); clarkfc@gmail.com (F.C.N.B.); pfreitas@ufc.br (A.L.P.F.)

**Keywords:** polysaccharide, gastric damage, ethanol, nitric oxide, hydrogen sulfide

## Abstract

The aim of the present study was to investigate the gastroprotective activity of a sulfated-polysaccharide (PLS) fraction extracted from the marine red algae *Gracilaria caudata* and the mechanism underlying the gastroprotective activity. Male Swiss mice were treated with PLS (3, 10, 30 and 90 mg·kg^−1^, *p.o.*), and after 30 min, they were administered 50% ethanol (0.5 mL/25 g^−1^, *p.o.*). One hour later, gastric damage was measured using a planimeter. Samples of the stomach tissue were also obtained for histopathological assessment and for assays of glutathione (GSH) and malondialdehyde (MDA). Other groups were pretreated with l-NAME (10 mg·kg^−1^, *i.p.*), dl-propargylglycine (PAG, 50 mg·kg^−1^, *p.o.*) or glibenclamide (5 mg·kg^−1^, *i.p.*). After 1 h, PLS (30 mg·kg^−1^, *p.o.*) was administered. After 30 min, ethanol 50% was administered (0.5 mL/25g^−1^, *p.o.*), followed by sacrifice after 60 min. PLS prevented-ethanol-induced macroscopic and microscopic gastric injury in a dose-dependent manner. However, treatment with l-NAME or glibenclamide reversed this gastroprotective effect. Administration of propargylglycine did not influence the effect of PLS. Our results suggest that PLS has a protective effect against ethanol-induced gastric damage in mice via activation of the NO/K_ATP_ pathway.

## 1. Introduction

Marine organisms are sources of numerous new compounds with multiple pharmacological properties [1]. At present, the number of substances being isolated from such sources is growing. Most of their chemical structures have been elucidated, and they are being investigated for their potential to meet various biological objectives, as well as to expand the scientific knowledge base in the area of naturally bioactive compounds [2]. Currently, about 25–30% of all active agents used in treatments are extracted from natural products [3]. In the last 50 years, sulfated-polysaccharide has drawn the attention of researchers, since it has become clear that it is involved in several cellular processes [4] and therefore, may present many pharmacological opportunities [5–8].

In algae, these sulfated-polysaccharides are complex macromolecular constituents of the extracellular matrix, and they evidently play an important role in the mechanical, osmotic, and ionic regulation of these beings [9,10]. Investigation of these biomolecules has been steadily increasing in recent years owing to their broad potential development as antithrombotic, anti-viral, anticoagulant, antioxidant, anti-inflammatory, and anti-proliferative agents [11–14]. However, studies of sulfated-polysaccharide extracted from sea algae (hereafter referred to as “PLS” throughout this manuscript) in ethanol-induced gastric damage models are scarce.

In the gastrointestinal tract, ethanol can produce acute hemorrhagic gastric damage, and excessive ingestion can result in gastritis characterized by mucosal edema, subepithelial hemorrhages, cellular exfoliation, and inflammatory cell infiltration [15,16]. Multiple mechanisms are likely to be involved in this pathogenic process, including depletion of non-protein sulfhydryl groups (thereby increasing reactive oxygen species (ROS) that have ulcerogenic activity), modulation of the nitric oxide system, and reduction of gastric mucosal blood flow. It has also been suggested that oxidative stress and depletion of anti-oxidants may be a crucial step in ethanol-induced mucosal damage [17]. In recent years, algal sulfated-polysaccharides have been demonstrated to play an important role as free-radical scavengers and antioxidants in the prevention of oxidative damage in living organisms [13,18].

Nitric oxide (NO) and hydrogen sulfide (H_2_S) are crucial mediators of gastrointestinal mucosal defense [19–22]. Studies have demonstrated that these gases are responsible for the modulation of some general components of mucosal defense such as increase of the gastric mucosal blood flow, mucus and bicarbonate secretion, and inhibition of neutrophil adherence to endothelial cells [19,23–25]. Thus, considering that the sea algae are important sources of new chemical substances with potential therapeutic effects, this study sought to evaluate the gastroprotective effect of a sulfated-polysaccharide fraction extracted from the sea algae *Gracilaria caudata* (PLS) against ethanol-induced gastric damage in mice, and the possible mechanisms involved.

## 2. Results and Discussion

**Figure 1:**
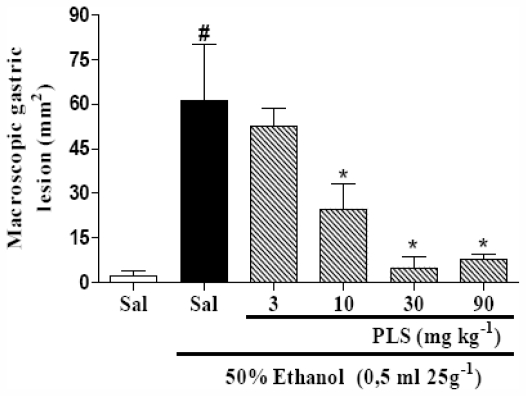
The effect of PLS on ethanol-induced gastric damage. Mice were treated by gavage with either saline or PLS (3, 10, 30 and 90·mg·kg^−1^). Thirty minutes later, mice in experimental groups were administered 50% ethanol (0.5 mL/25 g^−1^); the negative control group was administered saline. The total area of macroscopic gastric lesions was determined after 1 h. The results are expressed as mean ± SEM of a minimum of 5 animals per group. ^#^ *p* < 0.05 *vs.* saline group; * *p* < 0.05 *vs.* ethanol group; ANOVA and Newman-Keuls test.

Alcohol-related diseases of the gastrointestinal tract play an important role in clinical gastroenterology. However, the mechanisms and pathophysiology underlying the effects of ethanol on the organs of the digestive tract are not yet completely understood. The gastroprotective activity of PLS was evaluated using the ethanol-induced gastric damage model, the most commonly employed model in the evaluation of anti-ulcer/cytoprotective activity. Ethanol administration evidently resulted in severe macroscopic and microscopic gastric mucosal damage through an increase in reactive oxygen species generation and a decrease in the endogenous anti-oxidant defense mechanisms [26]. In the present study, we confirmed that ethanol induced gastrophathy (61.3 ± 18.9 mm^2^) and that PLS treatment reduced the macroscopic and microscopic ethanol-induced gastric damage. Figure 1 shows that the PLS prevented ethanol-gastropathy in a dose-dependent manner, reaching maximal effect at a dose of 30 mg·kg^−1^ (4.9 ± 3.7 mm^2^). Because a PLS dose of 30 mg·kg^−1^ afforded the most protection against gastric lesions induced by ethanol, this dose was selected for the study of the possible mechanisms of action involved in PLS-mediated gastroprotective effects.

**Figure 2:**
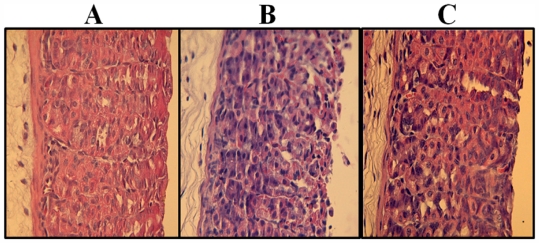
Photomicrographs of gastric mucosa (Magnification, 100×): (**A**) saline control; (**B**) animals treated with 50% ethanol, showing disruption of the superficial region of the gastric gland with epithelial cell loss and intense hemorrhage; (**C**) animals treated with 50% ethanol + polysaccharide (30 mg·kg^−1^), showing preservation of the gastric mucosa. Quantitative results from these assessments are shown in Table 1.

Figure 2 shows that ethanol administration induced a gastric superficial region disruption with epithelial cell loss and intense hemorrhage. Conversely, these afflictions were not observed in mice treated with ethanol and 30·mg·kg^−1^ of PLS. These data suggest that PLS has a gastroprotective effect in this context. Table 1 shows that PLS treatment (30 mg·kg^−1^) resulted in less ethanol-induced hemorrhagic damage, edema, and epithelial cell loss. Notably, these data indicate that ethanol did not increase inflammatory cell infiltration in the gastric mucosa compared with controls; however, this is probably due to mice being sacrificed just 1 h after ethanol administration (Figure 2 and Table 1).

**Figure 3:**
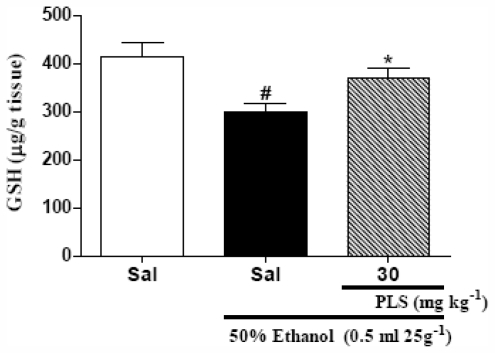
The effect of PLS on glutathione (GSH) levels in the gastric mucosa of mice treated with ethanol. Mice were treated by gavage with saline or PLS (30 mg·kg^−1^). Thirty minutes later, 50% ethanol (0.5 mL 25 g^−1^) was administered to the experimental groups, while the control group was administered saline. Ethanol administration promoted a reduction in the GSH gastric levels. This effect was partially reverted when the animals were treated with PLS. The results are expressed as mean ± SEM of 5 animals per group. ^#^ *p* < 0.05 *vs.* saline group; * *p* < 0.05 *vs.* ethanol group; ANOVA and Newman-Keuls test.

**Figure 4:**
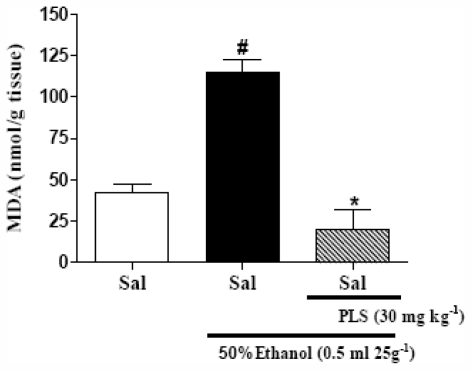
The effect of PLS on malondialdehyde (MDA) concentration in the gastric mucosa of mice treated with ethanol. Mice were treated by gavage with PLS (30 mg·kg^−1^). Thirty minutes later, mice in the experimental group were administered 50% ethanol (0.5 mL 25 g^−1^), and the control group was administered saline. The ethanol evidently promoted an increase in MDA gastric levels. When the animals were pre-treated with PLS, this effect was reverted. The results are expressed as the Means ± SEM of 5 animals per group. ^#^ *p* < 0.05 *vs.* saline group; * *p* < 0.05 *vs.* ethanol group; ANOVA and Newman-Keuls test.

It has been suggested that oxygen-derived free radicals (ROS) may contribute to ethanol-induced gastric mucosal lesions [27,28] and deplete the reduced glutathione (GSH) content in stomach tissues [21,27,29]. Our results are in accordance with these reports; ethanol induced a decrease in gastric GSH (298.3 ± 18.2 μg/g tissue) as compared to the saline group (415.0 ± 28.7 μg/g tissue) (Figure 3). Thus, PLS may function by decreasing the redox state in ethanol-induced gastropathy. Our results showed that administration of PLS (30 mg·kg^−1^; 369.7 ± 19.4 μg/g tissue) reversed the decrease in the gastric GSH levels after ethanol administration. Therefore, we inferred that the protective effect of PLS administration might be explained by a resultant increase in the gastric GSH concentration. Another possibility is that an increase in GSH levels could be secondary to a decrease in the free radical production. Superoxide produced by peroxidase in the stomach tissues might damage cell membranes and cause ulcers by increasing malondialdehyde (MDA) level, the most widely used index of lipid peroxidation [30]. Our results suggest that administration of 30 mg·kg^−1^ (20.0 ± 11.9 nmol·g^−1^ of tissue) of PLS resulted in a significant decrease in the MDA concentrations in ethanol-induced gastropathy (115.1 ± 7.4 nmol·g^−1^ of tissue) (Figure 4). Several authors evaluated the effect of different gastroprotective and antioxidants extracts, also had similar results [29–31]. Thus, the mechanism through which PLS exerts gastroprotective effects seems to involve an indirect antioxidant activities and a reduction of the lipid peroxidation induced by ethanol.

**Figure 5:**
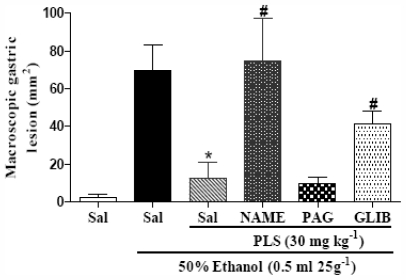
The effect of l-NAME, PAG, and glibenclamide on PLS-mediated protection against macroscopically visible ethanol-induced gastric damage. Mice were initially treated with l-NAME (10 mg·kg^−1^, *i.p.*), dl-propargylglycine (PAG, 50 mg·kg^−1^, *p.o.*), or glibenclamide (5 mg·kg^−1^, *i.p.*). After 1 h, PLS (30 mg·kg^−1^, *p.o.*) was administered. Thirty minutes later, 50% ethanol was administered to the experimental groups, and the control group was administered saline. The total area of the macroscopic gastric lesions was determined after 1 h. The results are expressed as mean ± SEM of a minimum of 5 animals per group. * *p* < 0.05 *vs.* ethanol group; ^#^ *p* < 0.05 *vs.* PLS + ethanol group; ANOVA and Newman-Keuls test.

Using a pharmacological approach it was demonstrated that PLS provides a protective effect against ethanol-induced gastric damage via the NO/K_ATP_ pathway. As shown in Figure 5, in animals treated with l-NAME (74.9 ± 22.9 mm^2^, a non selective inhibitor of nitric oxide synthase) or with glibenclamide (41.6 ± 6.5 mm^2^, a drug that blocks K_ATP_-dependent channels), the gastroprotective effect of PLS 30 mg·kg^−1^ (12.5 ± 8.3 mm^2^) was abrogated. This attests to the involvement of the NO/K_ATP_ pathway in the protection conferred by PLS. However, when the animals were pretreated with dl-propargylglycine (9.8 ± 3.5 mm^2^, an H_2_S synthesis inhibitor that blocks CSE activity), it did not alter the protective effect of PLS.

Nitric oxide (NO) modulates several elements of gastric mucosal defense, including blood flow [32], neutrophil adhesion [33,34], and mucus secretion [25,35]. NO and cyclic GMP can both activate various types of K^+^ channels [36,37]. Recently, it was demonstrated that the activation of ATP-sensitive potassium channels (K_ATP_) is involved in gastric defense [38,39]. Our findings suggest that the PLS defensive effect is a NO-dependent process and that blockage of the NO/K_ATP_ pathway with l-NAME/glibenclamide abrogated the protective effect of PLS against ethanol-induced gastric damage.

It has been demonstrated that the development of stress-induced gastric mucosal injury, including an increase in the gastric acid secretion, is involved in the pathogenesis of ethanol-induced gastric mucosal injury [40]. Several studies suggest that hypersecretion of gastric acid is associated with ethanol-induced gastric damage, and the anti-ulcer activity of many compounds may be related to the inhibition of gastric acid secretion [41,42]. However, in our study, PLS did not alter gastric acid secretion. As shown in Table 2, administration of PLS did not change the volume of gastric juice, the pH or the total acidity as compared to similarly derived values observed in the saline group. In contrast, histamine treatment increased the volume and total acidity, while ranitidine (an H_2_ antagonist) decreased the volume and total acidity, as compared to corresponding values in the saline group (Table 2).

In summary, our results indicate that PLS prevents ethanol-induced gastric damage. While there are many mechanisms through which this effect could potentially occur, our data supports the hypothesis that the reduction of lipid peroxidation and the activation of the NO/K_ATP_ pathway are of primary importance. These observations also raise the possibility of polysaccharides being used to improve resistance to gastric mucosal injury.

## 3. Experimental Section

### 3.1. Extraction of Soluble Polysaccharide

Specimens of the red algae *Gracilaria caudata* were collected in August 2008 from the Atlantic coast northeast of Brazil (Fleixeira Beach, Trairi-Ceará). After collection, the algae were cleaned of epiphytes, washed with distilled water, and stored at −20 °C. In order to enable extraction of polysaccharide, 5 g of dried *G. caudata* tissue was ground into a fine powder and incubated in stirring distilled water (1.5% w/v) for 2 h at 100 °C. After filtration and concentration of the solution, the polysaccharide was precipitated with ethanol (1:3 v/v) followed by washing with acetone and dried with hot air flow. The polysaccharide fraction was then re-dissolved in distilled water (1.5% w/v) and subjected to the same process to achieve precipitation, washing, and drying. The polysaccharide fraction thus derived constitutes what is referred to throughout this manuscript as “PLS”.

### 3.2. Animals

Male Swiss mice (25–30 g) were fasted for 18 to 24 h before the experiments. Animals were housed in cages in temperature-controlled rooms, and food and water were available *ad libitum*. All animal treatments and surgical procedures were performed in accordance with the Guide for Care and Use of Laboratory Animals (National Institute of Health, Bethesda, MD, USA) and were approved by the appropriate ethics committee (protocol No. 0066/10).

### 3.3. The Effect of PLS on Ethanol-Induced Gastric Damage

Male Swiss mice were initially treated with PLS (3, 10, 30 and 90 mg·kg^−1^) by gavage. After 30 min, gastric damage was induced in the experimental groups by ethanol administration (0.5 mL/25 g^−1^ *p.o.*), while the control group received saline. One hour later, the animals were sacrificed and their stomachs rapidly removed—opened via an incision along the greater curvature and pinned out on a wax block. Gastric damage (hemorrhagic or ulcerative lesions) was measured using a computer planimetry program (Image J^®^). A sample of the corpus region of each stomach was fixed in 10% formalin immediately after removal for subsequent histopathological assessment. Further gastric corpus samples were then weighed, frozen, and stored at −70 °C until they were assayed for GSH [43] and MDA [44].

### 3.4. The Roles of NO and H_2_S in the Protective Effect of PLS

Animals were pre-treated with a non-selective NO-synthase inhibitor, *N*-nitro-l-arginine methyl ester hydrochloride (l-NAME, 10 mg kg^−1^, *i.p.*), or with dl-propargylglycine (PAG, 50 mg·kg^−1^, *p.o.*), an inhibitor of H_2_S synthesis. After 1 h, the mice were administered PLS (30 mg·kg^−1^ *p.o.*). Thirty minutes later, gastric damage was induced in the experimental mice by intragastric instillation of ethanol 50% (0.5 mL/25 g^−1^ *p.o.*), while the control group received saline. One hour later, gastric damage was analyzed as described above.

### 3.5. The Role of K_ATP_ in PLS-Mediated Gastric Protection

To study the role of K_ATP_ in PLS-mediated gastric protection, animals were pre-treated with glibenclamide (5 mg·kg^−1^, *i.p.*), a drug that blocks K_ATP_-dependent channels. After 1 h, the mice received PLS (30 mg·kg^−1^ *p.o.*). Thirty minutes later, gastric damage was induced in experimental mice by intragastric instillation of ethanol 50% (0.5 mL/25 g^−1^ *p.o.*), while the control group received saline. One hour later, gastric damage was determined as described above.

### 3.6. Histological Assessment

For histological assessment, stomach samples were fixed in 10% formalin solution, sectioned, and embedded in paraffin. Four-micrometer-thick sections were deparaffinized, stained with hematoxylin and eosin, and then examined under a microscope. The specimens were assessed according to the criteria described in Laine and Weinstein (1988) [45]. In brief, 1 cm lengths of each histological section were assessed for epithelial cell loss (a score of 0–3), edema in the upper mucosa (a score of 0–4), hemorrhagic damage (a score of 0–4) and the presence of inflammatory cells (a score of 0–3). Subsequently, the sections were assessed as “blind” (without knowledge of the prior treatments) by an experienced pathologist.

### 3.7. GSH Assay

The reduced GSH content of the stomach tissues was estimated according to the method described in Sedlak and Lindsay (1968) [43]. A segment from each stomach was homogenized in 5 mL of cold 0.02 M EDTA solution (1 mL 100 mg^−1^ tissue). Aliquots (400 μL) of tissue homogenate were mixed with 320 μL of distilled water and 80 μL of 50% (w/v) trichloroacetic acid in glass tubes and centrifuged at 3000 rpm for 15 min. Next, 400 μL of each supernatant was mixed with 800 μL of Tris buffer (0.4 M, pH 8.9), and 20 μL of 0.01 M 5,5-dithio-bis (2-nitrobenzoic acid). After shaking the preparation, absorbance was measured at 412 nm on a spectrophotometer. GSH concentration was ascertained via a reduced GSH standard curve, generated in parallel. The results are expressed as micrograms of GSH per gram of tissue.

### 3.8. MDA Assay

The level of MDA in the homogenate from each group was measured using the method described in Mihara and Uchiyama (1978) [44], which is based on a thiobarbituric acid reaction. Fragments of the gastric mucosa weighing between 100 and 150 mg were homogenized with cold 1.15% KCl to prepare 10% homogenates. In brief, 250 μL of each homogenate was added to 1.5 mL of 1% H_3_PO_4_ and 0.5 mL of 0.6% *tert*-butyl alcohol (aqueous solution). Then, this mixture was stirred and heated in a boiling water bath for 45 min. The preparation was then cooled immediately in an ice water bath, followed by the addition of 4 mL of *n*-butanol. This mixture was shaken and the butanol layer was separated by centrifugation at 1200 g for 10 min. Optical density was determined to be 535 and 520 nm, and the optical density difference between the 2 determinations was calculated as the *tert*-butyl alcohol value. MDA concentrations are expressed as millimoles per gram of tissue.

### 3.9. Gastric Acid Secretion

The technique described in Shay *et al*. (1945) [46] was utilized in the current study. Firstly, pylorus ligature was performed under inhalation anesthesia. Then saline and PLS (30 mg·kg^−1^) were injected intraperitoneally. In another group, gastric acid secretion in pylorus-ligated mice induced by histamine (5 mg·kg^−1^) or ranitidine (5 mg·kg^−1^) via *i.p.* injection was tested. After 4 h, the animals were sacrificed by deep inhalatory anesthesia, their stomachs were opened, and the gastric content collected. The final volume and pH were directly determined after washing the mucosal side of the stomach with 2 mL of distilled water. Total acidity of the gastric juice was titrated with NaOH 0.01 N, using 2% phenolphthalein as an indicator.

### 3.10. Statistical Analysis

All values are expressed as mean ± SEM. ANOVA and the Student-Newman-Keuls test were used to determine the statistical significance of the differences between the groups. For histological assessment, the Kruskal-Wallis nonparametric test was used, followed by Dunn’s test for multiple comparisons. Differences were considered to be significant when *p* < 0.05.

## 4. Conclusions

In conclusion, our results indicate that PLS has a potential gastroprotective effect against ethanol-induced gastrophathy. In addition, the mechanism of PLS-mediated protection may be related to decreases in free radical production and lipid peroxidation.

Although there are many mechanisms through which this gastroprotective effect could occur, our data support the hypothesis that NO and the activation of K_ATP_ channels are of primary importance. These observations also raise the possibility of PLS being used to improve resistance to gastric mucosal injury.

## Figures and Tables

**Table 1:** The effect of PLS (30 mg·kg^−1^) on ethanol-induced microscopic gastric damage.

Experimental group (*n = 5*)	Hemorrhagic damage (score, 0–4)	Edema (score, 0–4)	Epithelial cell loss (score, 0–3)	Inflammatory cells (score, 0–3)	Total (score, 0–14)
Saline	0	0 (0–1)	0	0	0
Ethanol	3 (3–4)	3 (3–4)	2 (2–3)	0 (0–1)	8 (6–10)
Ethanol + PLS 30 mg·kg^−1^	1 (0–2) *	1 (0–1) *	1 (0–1) *	0	3 (0–3) *

Data shown are medians with minimal and maximal scores shown in parentheses. The Kruskal-Wallis nonparametric test, followed by Dunn’s test was used for multiple comparisons of histological analyses.

* *p* < 0.05 *vs.* control (ethanol) group.

**Table 2:** The effects of PLS on gastric secretion in 4-h pylorus-ligated mice.

Experimental Group (*n* = 5)	Volume (μL)	pH	Total Acid (mEq[H^+^]/L/4 h)
Saline	504 ± 39.2	1.6 ± 0.4	5.0 ± 0.3
PLS (30 mg·kg^−1^)	550 ± 89.1	1.8 ± 0.8	5.4 ± 0.6
Histamine	993 ± 90.8 *	1.5 ± 0.5	10 ± 0.6 *
Ranitidine	295 ± 78.3 #	2.7 ± 0.5	2.4 ± 0.5 #

Data shown are expressed as mean ± SEM (*n* = 5).

* *p* < 0.05;

# *p* < 0.05, *vs.* saline group; ANOVA and Newman-Keuls test.
